# Use of remote control in the intraoperative telemetry of cochlear implant: multicentric study^☆^

**DOI:** 10.1016/j.bjorl.2018.04.003

**Published:** 2018-05-18

**Authors:** Liege Franzini Tanamati, Maria Valéria Schmidt Goffi-Gomez, Lilian Ferreira Muniz, Paola Angélica Samuel, Gislaine Richter Minhoto Wiemes, Daniele Penna Lima, Sílvia Badur Curi, Lucia Cristina Onuki, Carla Fortunato Queiroz, Ana Karla Bigois Capistrano, Adriane Lima Mortari Moret, Márcia Yuri Tsumura Kimura, Valeria Oyanguren, Herbert Mauch

**Affiliations:** a Universidade de São Paulo (USP) – Campus Bauru, Bauru, SP, Brazil; b Universidade Federal de Pernambuco (UFPE), Recife, PE, Brazil; c Universidade de São Paulo (USP), Faculdade de Medicina, Hospital das Clínicas, São Paulo, SP, Brazil; d Universidade do Paraná (UFPR), Faculdade de Medicina, Hospital das Clínicas, Curitiba, PR, Brazil; e Hospital do Coração de Natal, Natal, RN, Brazil; f Universidade de Campinas (UNICAMP), Faculdade de Medicina, Hospital das Clínicas, Campinas, SP, Brazil; g Politec Saúde – Representante Cochlear no Brasil, São Paulo, SP, Brazil; h Cochlear Latin America, Panama City, Panama

**Keywords:** Cochlear implant, Impedance, Evoked potential, Telemetry, Implante coclear, Impedâncias, Potencial evocado, Telemetria

## Abstract

**Introduction:**

The conventional evaluation of neural telemetry and impedance requires the use of the computer coupled to an interface, with software that provides visualization of the stimulus and response. Recently, a remote control (CR220^®^) was launched in the market, that allows the performance of intraoperative tests with minimal instrumentation.

**Objective:**

To evaluate the agreement of the impedance values and neural telemetry thresholds, and the time of performance in the conventional procedure and by the remote control.

**Methods:**

Multicentric prospective cross-sectional study. Intraoperative evaluations of cochlear implants compatible with the use of CR220^®^ were included. The tests were carried out in the 22 electrodes to compare the time of performance in the two situations. The agreement of the neural telemetry threshold values obtained from five electrodes was analyzed, and the agreement of impedance was evaluated by the number of electrodes with altered values in each procedure.

**Results:**

There were no significant difference between the impedance values. There was a moderate to strong correlation between the electrically-evoked compound action potential thresholds. The mean time to perform the procedures using the CR220 was significantly lower than that with the conventional procedure.

**Conclusion:**

The use of the CR220 provided successful records for impedance telemetry and automatic neural response telemetry.

## Resumo

**Introdução:**

A avaliação convencional da telemetria neural e de impedâncias implica o uso do computador acoplado a uma interface, o *software* fornece o estímulo e a visualização das respostas. Recentemente, foi lançado um controle remoto (CR220^®^), que possibilita testes intraoperatórios com instrumental mínimo.

**Objetivo:**

Avaliar a concordância dos valores das impedâncias e dos limiares da telemetria neural e o tempo de execução no procedimento convencional e pelo controle remoto.

**Método:**

Estudo prospectivo transversal multicêntrico. Foram incluídas as avaliações intraoperatórias de implante coclear compatível com o uso do CR220^®^. Os testes foram realizados nos 22 eletrodos para comparar os tempos de execução nas duas situações. Foi analisada a concordância dos valores do limiar da telemetria neural obtidos em cinco eletrodos e a concordância das impedâncias foi avaliada pelo número de eletrodos com valores alterados em cada procedimento.

**Resultados:**

Não houve diferença significante entre as impedâncias. Obteve-se moderada a forte correlação entre os limiares do potencial de ação composto eletricamente evocado. O tempo médio para os procedimentos com o CR220 foi significativamente menor do que com o procedimento convencional.

**Conclusão:**

O uso do CR220 proporcionou registros bem-sucedidos para a telemetria de impedância e a telemetria automática de respostas neurais.

## Introduction

The use of objective data recording during the intraoperative procedure is part of the routine of cochlear implant programs worldwide. Such data are useful for analyzing device integrity, determining whether there are measurable neural responses, assisting in prognostic determination, contributing to the choice of speech processor programming parameters; they also provide an option to evaluate auditory nerve response alterations over time.^1^, ^2^, ^3^

The most commonly used intraoperative procedures are impedance telemetry and neural response telemetry. Impedance telemetry measures the opposition to the current flow, from the system characteristics, and its measurement allows the evaluation of the implanted system electronic integrity. High impedances can signify broken electrodes, absence of electrode–tissue contact, or electrodes located outside the cochlea. Low impedances may suggest electrodes in short-circuit. In any of these situations, electrodes with impaired impedances must not be activated or used to measure the neural response.^4^, ^5^

Neural response telemetry (NRT) is a rapid, noninvasive and objective measure of peripheral neural function. It is a measure derived from the electrical stimulation of the auditory nerve and the obtained response reflects the electrically-evoked compound action potential (ECAP). The responses have a relatively short latency, generally less than 0.5 ms. They are evoked using biphasic pulse currents and measured by an adjacent electrode that sends the response to the cochlear implant (CI) speech processor. The ECAP is characterized by a negative peak (N1) followed by a positive peak (P2), and the amplitude of the response between N1–P2 increases with increasing current level. The main advantage of measuring ECAP over the other measures of evoked potentials is that it can be recorded rapidly in CI users of any age, with no need for surface electrodes or sedation or even for the child to remain quiet.^6^

Typically, the tests are carried out by an experienced audiologist using the specific external device, equipment and software. Recently, Cochlear™ has introduced CR220^®^, a remote assistant, capable of performing such measurements that has the advantage that it does not require other equipment besides the external device. In addition to the audiologist, other professionals are also present in the operating room: doctors, anesthesiologist, scrub nurses, neurophysiologist, all with their workstations. Sometimes this dynamic can hinder circulation in the room and compromise the work performance. The use of a portable, wireless device that allows the same procedures to be carried out as in a larger workstation can be very useful in optimizing the physical space, as well as contributing to the best mobility around the place, while maintaining the same quality of responses.^7^

Tavartkiladze et al.^8^ have previously studied both technologies to measure intraoperative ECAP data and concluded that there is a significant reduction in the time used to measure the ECAP thresholds with the use of the remote device (CR120^®^). Although the test performance time with the remote assistant is shorter, it is necessary to know if the measures by both procedures are equivalent and if there are false-positive or false-negative results, requiring the use of the standard software for advanced ECAP threshold assessment.

Considering the work dynamics of the professionals involved in cochlear implant programs, this study aimed to evaluate the percentage of the implanted population that exhibits a recordable neural response from both procedures, the agreement between the impedance values and neural telemetry thresholds, and the intraoperative test performance time performed with the current version of the remote assistant CR220 and the Custom Sound^®^ EP software (CS EP^®^).

## Methods

This study was submitted to and approved by the Research Ethics Committee under protocol CAAE: 43236915.5.1001.0068 and approved under Opinion number 1.076.661/2015.

### Study characterization and site

This was a cross-sectional, multicenter study, involving seven cochlear implant centers located in four Brazilian states: São Paulo, Rio Grande do Norte, Paraná and Pernambuco. Of these, six centers are located in University Hospitals, and all of them are part of the Brazilian Unified Health System (SUS).

### Participant's selection criteria

No inclusion criteria were established for the age group, considering that the measures to be obtained and the study device should and can be assessed at any age. Thus, the study included children, adults and elderly individuals who underwent cochlear implant surgery from September 2014 to March 2015, and who received cochlear implants compatible with the remote control used in this study.

### Participants’ characterization

A total of 84 subjects, ranging in age from 2 to 83 years and time of auditory sensory deprivation of 5 to 672 months, participated in this study.

The hearing loss etiologies and the implanted internal components are described in Table 1. The CI 422 device was used in 54% of the sample and the rest (46%) received the CI 24RE CA (Table 1).

**Table 1:** Demographic data of the studied population with hearing loss etiology and model of the cochlear implant internal unit in the study population.

	Adults *N* (%)	Children *N* (%)	Total *N* (%)
*Etiology*			
Idiopathic	8 (9.5)	22 (26.2)	30 (35.7)
Progressive idiopathic	15 (17.8)	2 (2.4)	17 (20.2)
Meningitis	3 (3.6)	3 (3.6)	6 (7.1)
Genetic	0	4 (4.8)	4 (4.8)
Cytomegalovirus	1 (1.2)	3 (3.6)	4 (4.8)
Syndromes	3 (3.6)	1 (1.2)	4 (4.8)
Congenital	0	3 (3.6)	3 (3.6)
Rubella	2 (2.4)	1 (1.2)	3 (3.6)
VIII nerve hypoplasia	0	3 (3.6)	3 (3.6)
Otosclerosis	2 (2.4)	0	2 (2.4)
Hypoxia	0	2 (2.4)	2 (2.4)
Preterm birth	0	2 (2.4)	2 (2.4)
Traumatic	1 (1.2)	0	1 (1.2)

*Ototoxicity*	0	1 (1.2)	1 (1.2)
Toxoplasmosis	0	1 (1.2)	1 (1.2)
Mumps	1 (1.2)	0	1 (1.2)

*Internal unit model*			
CI 24RE CA	17 (20.2)	22 (26.2)	39 (46.4)
CI 422	19 (22.6)	26 (30.9)	45 (53.6)
Total	36 (42.8)	48 (57.1)	84 (100)

### Data collection procedures

**Figure 1:**
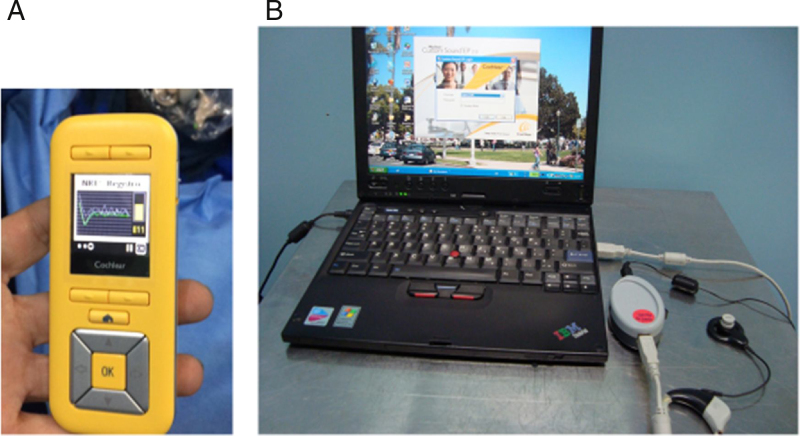
(A) Remote control unit called CR220^®^. (B) Set constituted by the software and programming interface was carried out by cable.

The following equipment was used to perform the proposed procedures (impedance telemetry and threshold assessment by automatic neural response telemetry – Auto NRT): the remote-control unit called CR220^®^ (Fig. 1A) and the Custom Sound^®^ EP Software, version 4.2, coupled to a connection interface.

Both procedures require a connection to the speech processor (Nucleus 5) for communication with the internal unit. The same processor was used in both test situations. Communication with the speech processor, using the wireless CR220^®^ and using the programming interface, was carried out through cable (Fig. 1B).

The software CS EP^®^ 4.2 was used for traditional data collection, i.e., without the aid of the remote unit. Although it did not require the use of software for data collection, the CR220^®^ needed to be coupled to a computer for the quantitative analysis of the obtained data, in addition to data storage.

The impedance telemetry was collected in all active and ground electrodes, and auto NRT was recorded for all 22 active electrodes, randomizing the collection order between the remote device (CR220^®^) and the standard equipment (computer + interface with CS EP^®^ 4.2 software).

The measurement/running time was collected from the moment the software was turned on to start the collection. The assembly and the connection of the interface and the speech processor were not computed for the standard procedure.

### Data analysis

**Figure 2:**
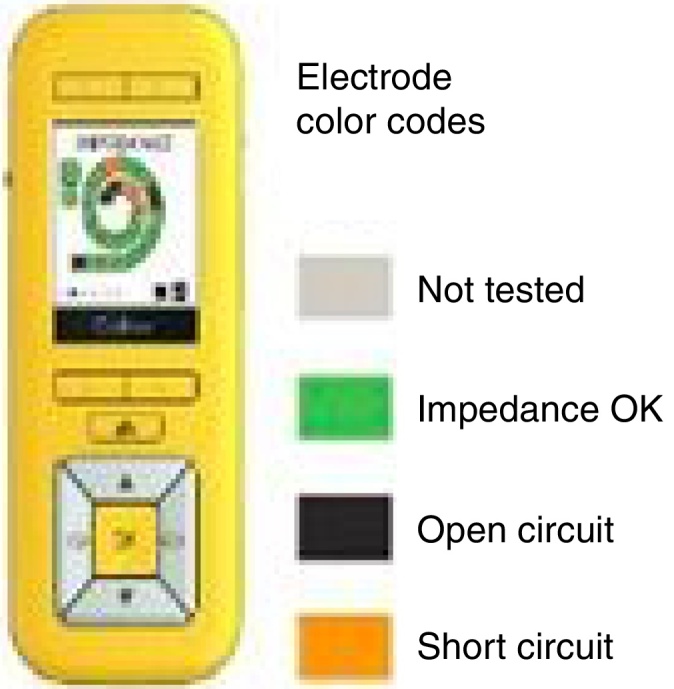
Information available for the remote assistant after evaluating the impedance and information on the electrode status.

Following the values proposed by the cochlear implant manufacturer, the impedance telemetry results were classified as^9^ (Fig. 2):

Normal: values between 0.565 kOhms (kΩ) and 30 kOhms (kΩ)
High impedances (HI): values >30 kOhms (kΩ)
Short circuit (SC): values <0.565 kOhms (kΩ)

After importing the data from the CR220^®^ to the Custom Sound^®^ software, the values were collected and a statistical comparison of the impedance values of electrodes E22, E16, E11, E6 and E1, expressed in kOhms, was performed between the two collection procedures.

The presence or absence of neural responses in all electrodes was evaluated in both procedures and the neural response threshold values, expressed as current units (cu), were compared between the collection procedures on electrodes E22, E16, E11, E6 and E1. Additionally, the tests’ measurement/performance time through CR220^®^ and Custom Sound^®^ EP were recorded and compared.

The paired *t*-test with *p* > 0.05 was used to compare the impedance levels; Pearson's correlation test was used for the comparison between ECAP thresholds and the paired *t*-test with *p* > 0.01 was used to compare the recording time of the procedures.

## Results

### Electrode impedance

Considering all 22 electrodes, the number of records obtained in the Impedance Telemetry test totaled 3690, of which 1845 were obtained through CR220^®^ and 1845, through CS EP^®^. Due to the partial insertion, three extracochlear electrodes were evaluated, which did not generate valid telemetry measurements. Thus, out of 3696 records, three measures of each condition (through CR220^®^ and through CS EP^®^) were excluded, totaling 3690 records. Of the 3690 records, 99.4% (3674 records) showed impedance values within the normal limits. Regarding the 16 records considered to be abnormal, 9 electrode SC records were obtained, as they showed impedance values below the normal range.

As for the other abnormal records, 7 HI electrode records were obtained, as they showed impedance values above the normal range. Table 2 shows the distribution of the modified impedance records per subject as a function of the measurement obtained through CR220^®^ and through CS EP^®^.

**Table 2:** Description of the electrode status, represented by the impedance values recorded by Custom Sound^®^ (standard procedure) and by the CR220^®^ (remote assistant).

	Custom Sound^®^ *n* (%)	CR220^®^ *n* (%)	Total *n* (%)
*Normal*	1837 (99.40%)	1837 (99.40%)	3674 (99.40%)

*High impedance (HI)*			7 (0.19%)
Coincident	3 (0.16%)	3 (0.16%)	
Discordant	1 (0.05%)	0 (0%)	

*Low impedance (SC)*			9 (0.24%)
Coincident	4 (0.22%)	4 (0.22%)	
Discordant	0 (0%)	1 (0.05%)	

*Extracochlear electrodes (partial insertion)*	3 (0.16%)	3 (0.16%)	6 (0.16%)

*Total*	1848 (100%)	1848 (100%)	3696 (100%)

SC, short circuit.

Regarding the abnormal impedance records, there was a discrepancy in the detection of a high impedance detected only through CS EP^®^, and a low impedance detected only through CR220^®^.

Regarding the impedance values measured in kOhms, there was no statistically significant difference between the values obtained through CR220^®^ and through CS EP^®^ (Table 3).

**Table 3:** Comparison of the impedance values (kOhms) recorded by Custom Sound^®^ (standard procedure) and CR220^®^ (remote assistant).

	Custom Sound^®^ Mean (SD)	CR220^®^ Mean (SD)	*p*
E1	8.70 (3.73)	8.34 (3.69)	0.562
E6	8.64 (3.88)	8.32 (3.75)	0.590
E11	8.81 (3.80)	8.56 (3.54)	0.695
E16	9.36 (3.88)	9.23 (3.46)	0.864
E22	12.38 (4.76)	11.67 (4.05)	0.336

SD, standard deviation.

*p* > 0.05 (paired *t*-test).

### Auto NRT

The neural response thresholds obtained in 5 electrodes were analyzed. Ten records corresponding to two patients could not be analyzed due to loss of data during the transfer from the CR220^®^ to the computer and three electrodes of the same patient were not measured because of a partial insertion, totaling 814 records, with 407 of them obtained during each procedure.

In the measurements obtained through CR220^®^, there were ECAP responses in 351 records, whereas in the CS EP^®^, there were ECAP responses in 361 records (Table 4, Table 5).

**Table 4:** Comparison of the presence of neural response recorded by Custom Sound^®^ (standard procedure) and by CR220^®^ (remote assistant).

	Custom Sound^®^ *N* (%)	CR220^®^ *N* (%)
Presence	361 (88.69)	351 (86.24)
Absence	46 (11.31)	56 (13.76)

**Table 5:** Comparison of the neural response thresholds (in current units – cu) recorded by Custom Sound^®^ (standard procedure) and by CR220^®^ (remote assistant).

	Custom Sound^®^ Mean (SD)	CR220^®^ Mean (SD)	*p*
E1	196.01 (21.88)	193.30 (19.54)	0.028
E6	196.68 (17.67)	194.99 (16.88)	0.016
E11	192.79 (14.01)	193.38 (14.68)	0.235
E16	184.65 (19.57)	184.34 (18.18)	0.249
E22	182.19 (20.74)	179.21 (15.39)	0.191

SD, standard deviation.

**Figure 3:**
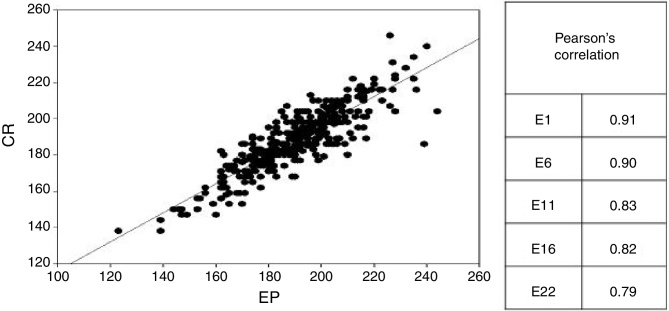
Correlation between ECAP thresholds measured through CR220^®^ and Custom Sound^®^ EP on all collected electrodes.

Regarding the ECAP thresholds, there was a moderate to strong correlation between the thresholds obtained through CR220^®^ and CS EP^®^ for the assessed electrodes E22, E16, E11, E6 and E1 (Fig. 3).

### Test performance time

The mean duration of the procedures (impedance telemetry and neural response telemetry) through CR220^®^ was 5.42 min and, for CS EP^®^ software, it corresponded to 7.39 min. This difference was statistically significant. The analysis obtained by device model also showed a statistically significant difference regarding the procedure performance time. For both Nucleus 422 and Nucleus Freedom (CI 24RE CA), procedure duration was significantly shorter when using CR220^®^ (Table 6).

**Table 6:** Mean time (in minutes) to perform the procedures through CR220^®^ and CS EP^®^ for the two electrode models (CI 422 and CI 24RE CA).

	CR220^®^	CS EP^®^
	Mean (SD) (in minutes)	
CI 422	5.20 ± 1.39^a^	7.75 ± 4.00
CI 24RE CA	5.68 ± 3.00^a^	6.98 ± 2.65

Total	5.42 ± 2.28^a^	7.39 ± 3.44

SD, standard deviation.

a *p* < 0.001 – statistically significant (paired *t* test).

## Discussion

Of the 84 participants in this sample, some had hearing loss caused by etiologies that could lead to cochlear ossification and, perhaps because of this, three electrodes of the same patient were not measured because there was a partial insertion, which reduced the number of records obtained in Auto NRT.

Another factor of result exclusion in this sample was the non-utilization of records of two patients due to loss of data to be analyzed in the CR220^®^ when transferred to the computer. Although the CR220^®^ is a device that is easy to handle and carry, it should be emphasized that it does not allow easy numerical visualization of data, which dictates the need to export the data for later analysis in a computer.

When analyzing the impedance telemetry, it was noted that there was no statistically significant difference between the values obtained through CR220^®^ and through CS EP^®^, when considering the impedance values of the electrodes measured in kOhms. However, some subjects showed electrodes in high impedance (HI) or short circuit (SC) and generally, in these situations, the altered electrodes detected through CR220^®^ were also detected through CS EP^®^, corroborating the concept of similar operation of the two devices regarding impedance telemetry performance. The reason for randomization of the order of evaluations in the intraoperative moment was because impedance values tend to decrease after the constant passage of current through the electrode.^10^ The discrepancy found in high impedance detection may be due to an air bubble that dissipated at retest with the second procedure.^5^ However, an electrode with altered impedance does not affect the course or conduct at the intraoperative moment and may be clinically circumvented at the speech processor programming.

Regarding the ECAP thresholds, in general, a moderate to strong correlation was found between the thresholds obtained through CR220^®^ and CS EP^®^ for the evaluated electrodes. The differences found for the RE CA were: in electrodes E1 and E6, they are not clinically representative, but these differences can be credited to differences in the algorithms used for the Auto NRT (expert system) in CS EP^®^ and in the CR220^®^ (decision tree). A study of 130 subjects using the same devices (CR220^®^ and CS EP^®^) to obtain ECAP showed a strong correlation (*r* = 0.90 and 0.97, respectively) between the results.^11^ The higher number of absent responses with the remote control could suggest that, in these situations, it would be necessary to confirm the neural response through the standard procedure that allows the use of advanced stimulation parameters, such as increase in pulse width or the number of events averaged.

When analyzing the recording time, the mean duration of the procedures (impedance telemetry and neural response telemetry) using the CR220^®^ was 5.42 min and, for the CS EP^®^ software, 7.39 min, with a statistically significant difference. The analysis obtained per device model, when comparing the tests in patients implanted with Nucleus CI 422 and with Nucleus Freedom (CI 24RE CA), the duration of the procedures was shorter when using CR220^®^, with a statistically significant difference in the time of the procedures.

Both technologies have been previously studied by Tavartkiladze et al.^8^ to measure intraoperative ECAP data. The authors collected the measures from 81 children and found that the mean time to measure the ECAP threshold using the Custom Sound^®^ software was 6.2 min (SD ± 1.0) vs. 4.8 min (SD ± 0.7) for the remote device (CR120^®^). The ECAP thresholds measured with Custom Sound^®^ software and CR120^®^ had a Pearson product-moment correlation coefficient for all electrodes of 0.92 (*p* < 0.01); with an absolute difference mean of 6 current units (SD ± 6). They concluded that there is a significant reduction in the time to measure ECAP thresholds with the use of the remote device (CR120^®^). Moreover, the threshold values measured with the CR120^®^ and the conventional software (Custom Sound^®^) were equivalent.

In a study carried out with 12 patients^12^ the authors obtained successful and uneventful records, with test duration of approximately 4 min. In a sample of 130 patients^13^ the mean time during the procedures corresponded to 7.4 min (SD = 1.9) and 5.4 min (SD = 1.0) for CS EP^®^ and CR220^®^, respectively. These data show that CR220^®^ provided a 22% decrease in intraoperative time when compared to the conventional method (*p* < 0.001). In our study, we did not evaluate the time of transference to the surgical center, or the waiting time, only the measurement time. However, Tavartkiladze et al.^11^ evaluated from the time of transfer, equipment preparation, waiting, measures, equipment storage and waiting time. For the authors, if we consider a clinic with 100 surgeries per year, the time the audiologist saved by utilizing the intraoperative test corresponds to the time occupied during 2 months in the clinic, instead of the going to, coming from and the unproductive wait in the operating room.

Humphries et al.^12^ and Olusesi et al.^13^ also pointed out that the remote control can be easily used by surgeons and assistants without the need for the audiologist to be present in the operating room.

The evaluation of the impedances and neural response thresholds are not the most important, but they are the only possible tests to be performed during the intraoperative moment.^6^, ^14^, ^15^, ^16^, ^17^ Therefore, the results of this study do not support the replacement of the Custom Sound^®^ EP by the CR220^®^, but they demonstrate an effective and fast alternative for the performance of basic tests during the Cochlear Implant surgery, in situations where it is not possible to have an audiologist in the operating room.

## Conclusion

The use of the CR220^®^ provided successful records for impedance telemetry and automatic neural response telemetry.

There was no statistically significant difference between the impedance values obtained through CR220^®^ and CS EP^®^ 4.2.

Regarding the Auto NRT, there was a moderated to strong correlation between the ECAP thresholds obtained through CR220^®^ and through CS EP^®^. A higher number of records showing the absence of ECAP were found for the CR220^®^. The mean time to perform the procedures using the CR220^®^ was significantly lower than with Custom Sound^®^ EP.

## Conflicts of interest

The authors declare no conflicts of interest and that they have not received any monetary bonuses, aid or fees involved with the study.

Authors Valeria Oyanguren, Herbert Mauch and Marcia Kimura have an employment relationship with the cochlear implant company responsible for the technology used in the study. Author Maria Valéria Schmidt Goffi-Gomez provides technical consulting services to the same company.
